# New pathway for the formation of metallic cubic phase Ge-Sb-Te compounds induced by an electric current

**DOI:** 10.1038/srep21466

**Published:** 2016-02-23

**Authors:** Yong-Jin Park, Ju-Young Cho, Min-Woo Jeong, Sekwon Na, Young-Chang Joo

**Affiliations:** 1 Department of Materials Science & Engineering, Seoul National University, Seoul 151-744, Korea; 2 Research Institute of Advanced Materials (RIAM), Seoul National University, Seoul 151-742, Republic of Korea

## Abstract

The novel discovery of a current-induced transition from insulator to metal in the crystalline phase of Ge_2_Sb_2_Te_5_ and GeSb_4_Te_7_ have been studied by means of a model using line-patterned samples. The resistivity of cubic phase Ge-Sb-Te compound was reduced by an electrical current (~1 MA/cm^2^), and the final resistivity was determined based on the stress current density, regardless of the initial resistivity and temperature, which indicates that the conductivity of Ge-Sb-Te compound can be modulated by an electrical current. The minimum resistivity of Ge-Sb-Te materials can be achieved at high kinetic rates by applying an electrical current, and the material properties change from insulating to metallic behavior without a phase transition. The current-induced metal transition is more effective in GeSb_4_Te_7_ than Ge_2_Sb_2_Te_5_, which depends on the intrinsic vacancy of materials. Electromigration, which is the migration of atoms induced by a momentum transfer from charge carriers, can easily promote the rearrangement of vacancies in the cubic phase of Ge-Sb-Te compound. This behavior differs significantly from thermal annealing, which accompanies a phase transition to the hexagonal phase. This result suggests a new pathway for modulating the electrical conductivity and material properties of chalcogenide materials by applying an electrical current.

Cubic phase Ge-Sb-Te compounds (GST), the most common phase change materials, have a high degree of disorder with large numbers of structural vacancies in their lattices^1^,^2^,^3^. This structural feature of the metastable cubic phase leads to the electrical properties and the charge transport properties being significantly influenced by temperature.

It was observed that the resistance of GeSb_2_Te_4_ gradually decreases with temperature, unlike GeTe, and that the characteristics change from insulator to metal at high temperatures demonstrated by T. Siegrist *et al.*^4^ The two types of GST can be distinguished by the temperature coefficient of resistivity (TCR) which has a positive value for metallic materials and a negative value for insulating materials. Theoretical studies using density functional theory calculations by W. Zhang *et al.* provided the microscopic origin of the metal-insulator transition (MIT) in cubic GST; the rearrangement of vacancies leads to a decrease in the total energy per atom, which implies that the arrangement of vacancies is critical in the electrical properties of cubic GST^5^. More recently, the impact of stoichiometry was demonstrated by P. Jost *et al.* as a controllable parameter for the MIT of the most common compositions in the (GeTe)_x_-(Sb_2_Te_3_)_1−x_ system; i.e., disorder-induced localization results in the MIT differ according to the average vacancy concentration of various compositions^6^. Therefore, the behaviors of the MIT controlled by the annealing temperature and the stoichiometry is critical for tailoring the charge transport properties of cubic GST to increase the performance of phase-change random access memory (PcRAM) devices.

Although the rearrangement of vacancies in the GST is considered to be the origin of MIT, it is difficult to separate the vacancy effect from phase transition because GST has two phases types: an insulating cubic phase and a metallic hexagonal phase^2^. The phase transition from the cubic to the hexagonal phase is a high priority before the rearrangements of vacancies can be completed^4^,^7^. Therefore, the final product of GST after MIT is the hexagonal phase of GST, and the vacancy effect on MIT has not been proven experimentally. Here, we investigated the driving force for MIT beyond annealing temperature or stoichiometry and independent from phase transition: electric current. An electric current has the power to migrate atoms and vacancies via momentum transfer, which is known as electromigration (EM)^8^. The atomic displacement induced by an electric current can promote the rearrangement of vacancies. It is worth investigating MIT under electric current stressing conditions, i.e., more device-related characteristics. During the RcRAM operation (writing, erasing and reading), the cubic GST is exposed to various ranges of electric current stressing, and there is much evidence of changes in the structural and charge transport properties under these circumstances^9^,^10^,^11^.

The electric current effect on crystalline GST has been typically associated with a high current density of over 10^7^ A/cm^2^ 10,11,12,13,14. In this condition, electric current generates disorder or massive displacement of atoms because the driving force for migration is sufficient to create structural changes with high current density. For instance, the elements of Ge_2_Sb_2_Te_5_ are demixed by an electric field, which causes void formation and compositional change^11^,^12^. Disorder can be induced by high current density in a nanowire system, leading to the amorphous or insulating phase^10^,^15^. In contrast, a relatively low current density of approximately 10^6^ A/cm^2^ cannot generate the structural disorder need to transition to the insulating phase. The atomic displacement by a low current is negligible compared with that from a high current because the driving force is insufficient to generate a massive flux of atoms^9^. An electric current in the range of 10^6^ A/cm^2^ only assists in the rearrangement of vacancies.

In this study, we demonstrate the transition from insulator to metal without phase transition using an electric current in crystalline GST, which differs from thermal annealing. This is a new pathway for metallic cubic phase formation; it has not been previously reported because GSTs generally have low resistivity in the hexagonal phase. The metallic cubic phase of GST is investigated by temperature dependency and microstructural analyses. Moreover, the influence of intrinsic vacancies on MIT was investigated by comparing Ge_2_Sb_2_Te_5_ (GST225) and GeSb_4_Te_7_ (GST147), which have structurally different initial vacancies^16^,^17^. Line-patterned samples were employed to study the current-induced transition in crystalline GST.

## Results

**Figure 1:**
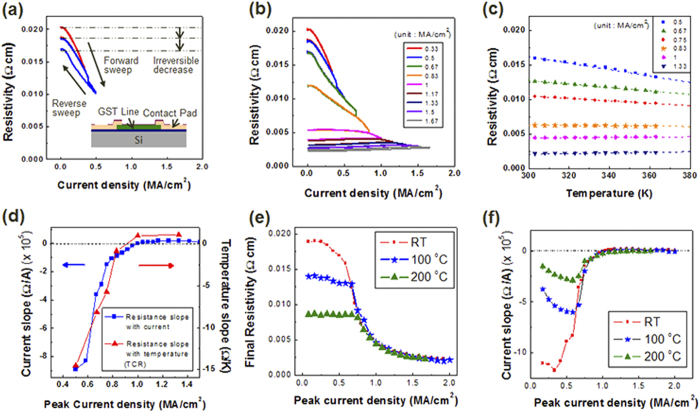
(**a**) Resistivity of Ge_2_Sb_2_Te_5_ (GST225) for a current sweep using the double directional mode, with a sweep from zero to peak current and a return to zero, at room temperature for the 0.33 and 0.5 MA/cm^2^ conditions. The inset figure displays a schematic image of the sample which shows the cross-sectional structure. (**b**) Resistivity change for peak current densities ranging from 0.33 to 1.67 MA/cm^2^ in increments of 0.17 MA/cm^2^. (**c**) The resistivity of current-stressed GST225 from (**b**) for temperatures ranging from 30 to 90 °C. The dotted lines are extrapolations whose slopes correspond to the temperature coefficient of resistance (TCR). (**d**) Resistivity slope for current density and temperature according to the stress peak current density results from (**b,c**). The behavior of the TCR was similar to the resistivity slope for the current density, which changed from negative to positive at the peak current density of 1 MA/cm^2^. (**e**) Final resistivity change of GST225 for peak current densities ranging from 0.17 to 2 MA/cm^2^ in increments of 0.08 MA/cm^2^ at different temperatures: room temperature, 100 °C, and 200 °C. (**f**) Slope of the resistivity of GST225 according to the peak current density at various temperatures based on the results from (**d**) (See supplementary information). The final resistivity and slope are independent of temperature.

Figure 1(a) shows the electrical resistivity of GST225 after a current sweep with an increasing peak current density of 0.33 and 0.5 MA/cm^2^ at room temperature. The double directional mode, with a sweep from zero to peak current and a return to zero, was used, and the peak current density increased each cycle from 0.33 to 1.67 MA/cm^2^ in 0.17 MA/cm^2^ increments, as shown in Fig. 1(b). As shown in Fig. 1(a), the resistivity of GST225 decreased with increasing current density, and a certain amount of the resistivity was unrecovered during the reverse sweep; i.e., an irreversible change in resistivity was observed. In contrast, the change in resistivity during the reverse sweep was the same as that for the forward sweep under the experienced current density; i.e., a reversible change in resistivity was displayed. These results indicate that an irreversible change in resistivity was completed in the first cycle of applied current. The slopes of the resistivity and current density during the reverse sweep increased with the peak current density, and their signs changed from negative to positive at a peak current density of 1 MA/cm^2^. Figure 1(c) shows the resistivity of GST225 for temperatures ranging from 30 to 90 °C after current stressing. A linear relationship between resistivity and temperature was observed, and their slopes decreased with increasing applied current. The slope of the resistivity and temperature gives the temperature coefficient of resistance (TCR), which is used to distinguish metallic (positive relationship) from insulating or disordered metal called “bad metal” (negative relationship) behavior^4^,^18^. The TCR of 0.83-MA/cm^2^-stressed GST225 (yellow star) was – 4.0 × 10^4^ 1/K, and the TCR of 1-MA/cm^2^-stressed GST225 (orange diamond) was 6.7 × 10^4^ 1/K, as shown in Fig. 1(c). The tendency of resistivity and temperature was confirmed upto 150 K. (see supplementary information, Fig. S6) Although the cubic phase of GST225 is known as the insulator, the negative relationship of TCR can be classified as a metal with weak localization in our results. The exact classification of the material properties through near 0 K will be discussed in the further study. Figure 1(d) shows the resistivity slope as a function of the current density, corresponding to the results in Fig. 1(b), and as a function of the temperature, corresponding to the results in Fig. 1(c), according to the stress peak current density. The slopes of resistivity according to current density during the reverse sweep and temperature are denoted as current slope and temperature slope, respectively. As shown in Fig. 1(d), the behavior of the TCR was similar to the resistivity slope for the current density, which changed from negative to positive at the peak current density of 1 MA/cm^2^. Because resistivity change in reverse sweep has a relationship with temperature, reversible change of resistivity under experienced condition is affected by Joule heating. Therefore, irreversible decrease of resistivity was completed in the first cycle of applied current, while reversible change of resistivity under experienced current density was affected by Joule heating, which is similar with the TCR measurement. Consequently, the current-stressed GST225 was regarded as displaying metallic behavior at current densities above 1 MA/cm^2^.

However, the above results can be explained by thermal effect of Joule heating even the irreversible decrease of resistivity. To distinguish the thermal from the electrical effect on the resistivity behavior, electric current was applied to GST225 with various ambient temperature. Figure 1(e) illustrates how the final resistivity of GST225 depends on each current sweep for various temperatures: room temperature, 100 °C, and 200 °C. Figure 1(f) shows the slope of the resistivity for each current sweep (for the results of the current sweep at various temperatures, see the Supplementary information, Fig. S2). As the temperature increased, the starting point of the resistivity decreased, as depicted in Fig. 1(e). However, the resistivity for a current density over 0.7 MA/cm^2^ showed the same results in the resistivity curve regardless of the temperature. Additionally, the sign of the resistivity slope changed from negative to positive at 1 MA/cm^2^, independent of the temperature, as shown in Fig. 1(f). This result implies that the resistivity of GST225 is only determined by the current density, regardless of the initial resistivity or external temperature. The crystalline GST had a fixed value of resistivity depending on the applied current density, and the process of resistivity change was irreversible. Therefore, once the initial resistivity was reduced by the temperature, the current density had no direct effect on the final resistivity until it reached the current density for the corresponding reduced resistivity. When the current density reached the condition of the corresponding resistivity, the resistivity followed the universal curve of GST225, which is useful for a resistivity-based device because the resistivity can be controlled by an electric current.

**Figure 2:**
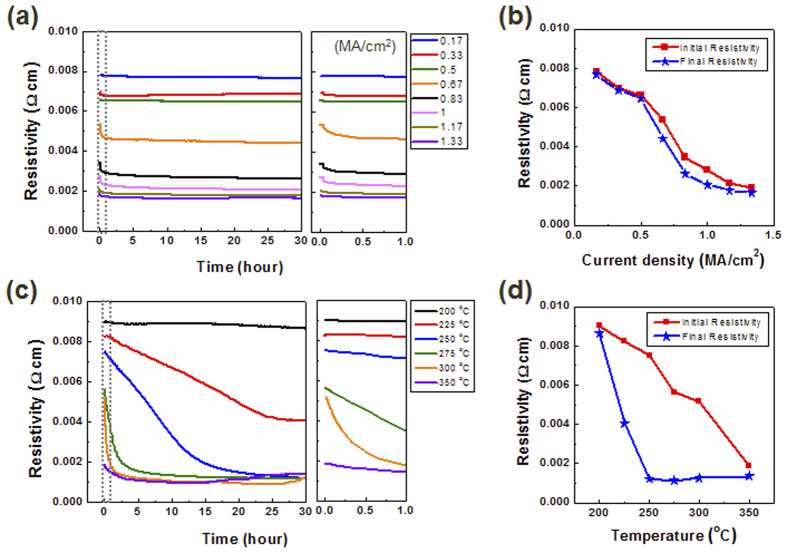
(**a**) Resistivity change of GST225 according to the duration of applied current density, ranging from 0.17 to 1.33 MA/cm^2^, for 30 hours and its expansion (dotted box) from 0 to 1 hour. (**b**) Initial and final resistivities of GST225 according to the current density for the current-stressed condition result from (**a**). (**c**) Resistivity change of GST225 according to the duration of applied temperature, ranging from 200 to 350 °C, for 30 hours and its expansion (dotted box) from 0 to 1 hour. (**d**) Initial and final resistivities of GST225 according to the ambient temperature for the isothermal annealing condition result from (**c**). The electric current has a kinetically fast effect on resistivity compared with thermal stress, which involves a thermally activated change.

To understand the kinetic properties of current-induced resistivity change in GST225, we investigated the influence of the current duration on the resistivity of GST225, as shown in Fig. 2(a). The resistivity of GST225 decreased with the application of a current density, but the change only occurred in the early phase of current stressing, similar to the results in Fig. 1(a). Figure 2(b) shows the initial and final resistivities of GST225 with an electric current applied for 30 hours, according to the current density. As mentioned above, a current-induced resistivity change occurred during the first cycle of the current sweep, indicating that the irreversible change in resistivity occurred within a few seconds. After this drastic change, the resistivity of GST225 remained unchanged for a long period, regardless of the current density. To compare this influence with that of electrical and thermal stress on the resistivity change in GST225, we also investigated resistivity according to the time annealed at various temperatures, as shown in Fig. 2(c). The resistivity of GST225 under isothermal annealing decreased with temperature, and a continuous change was observed for a long period of time, as shown in Fig. 2(c). The saturated resistivity, the time-independent point of resistivity, was inversely proportional to the ambient temperature. Fig. 2(d) shows the initial and final resistivities of GST225 (based on Fig. 2(c)) according to the temperature. Unlike the results for an applied current, significant differences were observed between the initial and final resistivities in the annealed condition as shown in Fig. 2(d). These results show that the electric current has a kinetically fast effect on the resistivity compared with thermal stress, which involves a thermally activated change. Although electrical and thermal stress have different time-dependent behaviors, they exerted a similar influence on the resistivity of GST225 to reach the minimum state of resistivity. For an electrical stress of 1 MA/cm^2^ and a thermal stress of 250 °C, the resistivity was saturated and unchanged. The current-stressed sample for 1 MA/cm^2^ and the temperature-stressed sample for 250 °C are denoted as the “current sample” and “temperature sample”, respectively.

**Figure 3:**
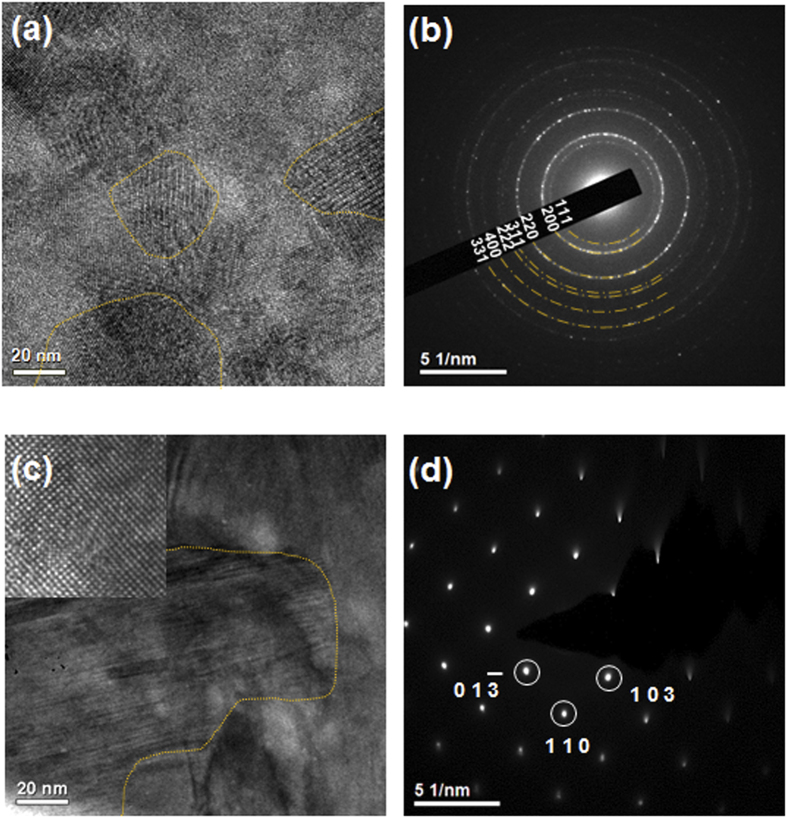
(**a**) TEM image and (**b**) diffraction pattern of the current-stressed GST225 sample (Current sample: 1 MA/cm^2^ condition). The yellow dotted lines indicate the grain boundary. The microstructure of the current sample was in a polycrystalline cubic phase and the average grain size was approximately 20 nm. (**c**) TEM image and (**d**) diffraction pattern of the temperature-stressed GST225 sample (temperature sample: 250 °C condition). An HRTEM image of a single grain is displayed in the inset of (**c**). Large grains of more than 100 nm in a hexagonal phase were observed in the temperature sample.

For a more detailed examination of the differences between the electrical and thermal effects, microstructural analyses were conducted. Figure 3 shows the microstructural image and diffraction pattern of a current and temperature sample of GST225. As shown in Fig. 3(a), the microstructure of the current sample was in a polycrystalline state and the average grain size was approximately 20 nm, as indicated by the dotted line. Due to the polycrystalline state and small grain size, a ring-type diffraction pattern was observed, as shown in Fig. 3(b). From the radius of the concentric circles, the crystalline phase of the current sample was confirmed as the face-centered cubic (FCC) phase of GST225. To represent entire phase, microstructural analyses were conducted in various positions, and the results were independent with position. (See supporting information, Fig. S7) In contrast, large grains of more than 100 nm were observed in the temperature sample, as shown in Fig. 3(c). High temperature caused an increase in the grain size of GST225. Figure 3(d) shows the diffraction pattern for Fig. 3(c), which is a spot-type pattern because of the large grains. Based on the distance (3.05 Å) and angle of the spots (87.5°), the crystalline plane of the temperature sample is determined to be the (013) family, and the phase was confirmed as the hexagonal close packing (HCP) phase of GST225. The initial sample, unstressed with current and temperature, was not a fully crystalline phase but had a grain size of approximately 20 nm and was in the cubic phase of GST225, similar to the current sample. Based on the standard phase of GST225 at room temperature, the only microstructural change in the current sample was crystallization. The temperature sample, however, had large grains and a different crystalline phase compared with the initial state. Interestingly, the current and temperature samples exhibited similar electrical resistivities, although the cubic phase displayed insulating behavior, whereas the hexagonal phase displayed metallic behavior. The current sample had a low resistivity, similar to the hexagonal phase, while maintaining the cubic phase. Therefore, the crystalline GST possesses another primary factor for the determination of resistivity in addition to the phase.

**Figure 4:**
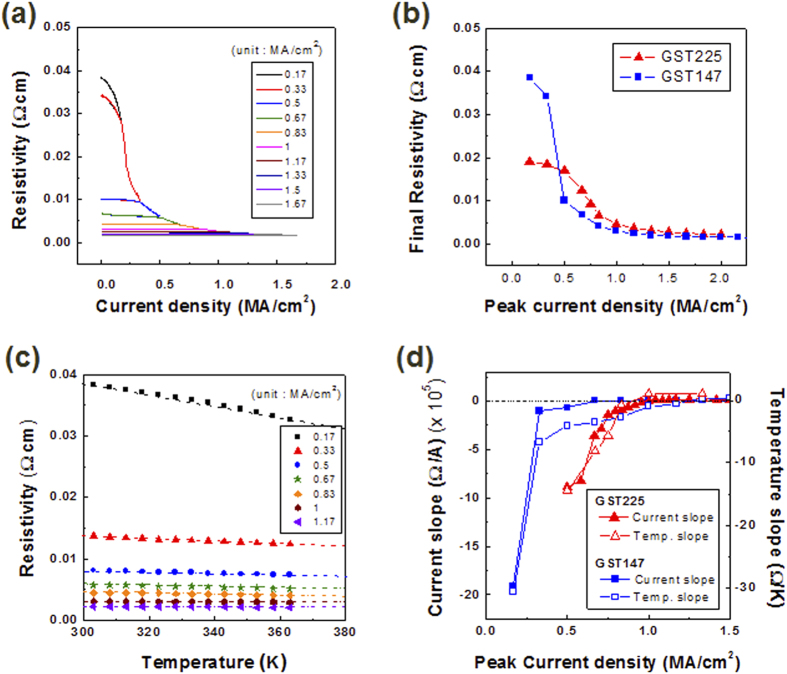
(**a**) Resistivity change of GeSb_4_Te_7_ (GST147) with current densities for peak current densities ranging from 0.17 to 1.67 MA/cm^2^ in increments of 0.17 MA/cm^2^ at room temperature. (**b**) Final resistivity of GST225 and GST147 according to peak current density at room temperature (results from Fig. 1(b) and Fig. 4(a), respectively). (**c**) The resistivity of current-stressed GST147 from (**a**) for temperatures ranging from 30 to 90 °C. The dotted lines are extrapolations whose slopes correspond to the TCR. (**d**) Resistivity slope of GST225 and GST147 for current density and temperature according to the stressed-peak-current-density results for GST225 (Fig. 1(b,c)) and GST147 (Fig. 4(a,c)). These results indicate that current-induced resistivity change was more effective in GST147 compared with GST225, which depends on the intrinsic vacancy of materials.

Because crystalline GST225 has a high number of intrinsic vacancies, the electrical properties of GST225 are governed by disorder between the constitutional atoms and vacancies^4^. To investigate the effect of intrinsic vacancy, similar experiments were conducted for GST147 which has more vacancies than GST225^16^,^17^. Figure 4(a) shows the electrical resistivity of GST147 after a current sweep with an increasing peak current density from 0.17 and 1.67 MA/cm^2^ in 0.17 MA/cm^2^ increments at room temperature. As shown in Fig. 4(a), the resistivity of GST147 decreased with increasing current density, and an irreversible change in resistivity was observed, which are similar to the results for GST225, as shown in Fig. 1(b). Figure 4(b) shows the final resistivity results for GST225 from Fig. 1(b) and the GST147 results from Fig. 4(a), according to the peak current density at room temperature. Although the resistivity of GST225 and GST147 decreased with peak current density, the degree and speed of decline in GST147 was much higher than that of GST225. These results indicate that electric current has a similar effect on GST147, but GST147 is much more sensitive to electric current than GST225. Figure 4(c) shows the resistivity of GST147 at temperatures ranging from 30 to 90 °C after current stressing. The slopes of resistivity and temperature, TCR, increased with peak current density from negative to near zero. The slope of resistivity with current density and temperature, according to the stress current density, for GST225 and GST147 are shown in Fig. 4(d), corresponding to the results for GST225 (Fig. 1(b,c)) and GST147 (Fig. 4(a,c)). As shown in Fig. 4(d), for GST225, the behavior of the TCR was similar to the resistivity slope for the current density, which changed from negative to positive at the peak current density of 1 MA/cm^2^. In the case of GST147, although the current and temperature slopes for GST147 have some differences, a similar slope change was observed and approached zero. Moreover, the current and temperature slopes for 0.33-MA/cm^2^-stressed GST147 were extremely changed compared with 0.17-MA/cm^2^-stressed GST147 despite differing by only 0.17 MA/cm^2^. These results indicate that the current-induced resistivity change was more effective on GST147 than GST225, which depends on the intrinsic vacancy of materials.

## Discussion

W. Zhang *et al.* calculated the total energy per atom for cubic and hexagonal GST with respect to vacancy ordering^5^. The rearrangement of vacancies affects the degree of disorder and creates a vacancy plane in both the cubic and hexagonal phases, which is the most stable state. However, the phase transition from the cubic to hexagonal phase is a high priority before the vacancy planes can be completely formed^4^. Therefore, GST225 has a low resistivity in the hexagonal phase, which agrees with our results for the temperature sample, as shown in Fig. 3(d). Surprisingly, the current-stressed GST225 has a low resistivity, similar to the hexagonal phase, while maintaining the cubic phase. An applied current can migrate atoms by momentum transfer from the charge carriers, which is known as EM^8^. The vacancy rearrangement is not triggered by electrical current, because the vacancy rearrangement is thermodynamically spontaneous process^5^. The role of electrical current is a kinetic factor which accelerate rearrangement process. Although the driving force of migration is too low to generate a massive flow of atoms for sub-MA/cm^2^ current densities, vacancy rearrangements can be easily promoted by EM. Therefore, both vacancy rearrangements and phase transitions involve a process of atomic arrangement. Phase transitions, however, require more energy to generate a new phase compared with vacancy rearrangements because phase transitions are accompanied by a global change in atoms. Because GST line was directly patterned on bulk Si, generated heat was spread out easily caused by high thermal conductivity and heat capacity of Si. Furthermore, resistivity of GST independent with frequency and voltage under alternating current (AC) condition unlike the direct current (DC) condition. (See supplementary information, Fig. S5) Because the joule heating of AC and DC should produce the same effect, this result indicates that the resistivity decrease is related to the bias dependence during current stressing. Therefore, a current can accelerate vacancy rearrangements without a phase transition.

In summary, we investigated the resistivity of GST225 and GST147 under an applied electric current for a range of temperatures. The resistivity is determined by the current density, regardless of the initial resistivity. Without a phase transition, the minimum resistivity can be achieved kinetically fast by applying a current density, which differs significantly from thermal annealing. This phenomenon depends on the initial vacancy of materials. This work provides a new pathway for promoting vacancy rearrangements using an electric current and deepens our understanding of the material physics of phase-change materials.

## Method

### Device fabrication

Line-patterned GST samples, which were 20 μm in length, 2 μm in width and 300 nm in thickness^9^, were subjected to an electric current or various temperatures. Thin films of GST were deposited using a DC magnetron sputterer on a 10-nm silicon oxide wafer and were patterned by photolithography using an aligner (Karl-Suss MA-6 II). To prevent evaporation and oxidation of the GST, a 100-nm layer of silicon nitride was deposited on the GST lines. A schematic of the sample is shown in the inset of Fig. 1a, which shows the cross-sectional structure of the sample. The pre-annealing process is used to crystallize the amorphous phase of the as-deposited GST into the crystalline cubic phase for 1 hour at 200 °C.

### Electrical measurement

I-V characteristics of the GST samples were measured using an Agilent 4156C parameter analyzer with a current ranging from 0.17 to 2 MA/cm^2^ in increments of 0.17 MA/cm^2^. The substrate temperatures were varied from RT to 200 °C. Long-term stressing was applied to the GST samples using a packaged-level electromigration tester (QualiTau MIRA), with currents ranging from 0.17 to 1.33 MA/cm^2^ and temperatures ranging from 200 to 350 °C.

### Microscopic analysis

The cross-sectional image of the current and temperature samples were taken using a transmission electron microscopy (TEM, Tecnai F20). The TEM samples were fabricated using a focused ion beam (FIB, SII NanoTechnology SMI3050SE). Microstructural analysis using X-ray diffractometry (XRD, PANalytical X’pert Pro) was also performed.

## Additional Information

**How to cite this article**: Park, Y.-J. *et al.* New pathway for the formation of metallic cubic phase Ge-Sb-Te compounds induced by an electric current. *Sci. Rep.* **6**, 21466; doi: 10.1038/srep21466 (2016).

## Supplementary Material

Supplementary Information
