# Supplementary material for: New pathway for the formation of metallic cubic phase Ge-Sb-Te compounds induced by an electric current
Source: Sci Rep. 2016 Feb 23;6:21466. doi: 10.1038/srep21466 (PMC4763274; doi:10.1038/srep21466)
Supplement: Supplementary Information [file srep21466-s1.doc]

Supplementary Information

**New pathway for the formation of metallic cubic phase Ge-Sb-Te compounds induced by an electric current**

Yong-Jin Park, Ju-Young Cho, Min-Woo Jeong, Sekwon Na, and Young-Chang Joo*

**1. Resistivity change behavior during cyclic current sweep in GST225**

A cyclic current sweep was performed with a fixed peak-current density of 1.67 MA/cm^2^ for GST225. Fig. S1 shows the resistivity of GST225 during a current sweep from 0 to 1.67 MA/cm^2^ at room temperature 10 times, and the inset of Fig. S1 shows a range of resistivity from 0.0018 to 0.0035 Ωcm. The first sweep (black line in Fig. S1) shows a continuous decrease in resistivity to 1.67 MA/cm^2^ during the foward sweep and a slight change in resistivity during the reverse sweep to zero. The second to tenth sweeps, however, follows the reverse sweep of the first sweep with some variation. Furthermore, the resistivity for a direct sweep to 1.67 MA/cm^2^ (Fig. S1(a)) is the same as the resistivity for sequential sweeps to 1.67 MA/cm^2^ increasing in increments of 0.17 MA/cm^2^ (Fig. 1(b)). These results indicate that an irreversible change in resistivity was completed during the first cycle of applied current. Once irreversibly changed, the resistivity was resversible under the experienced current density.

**2. The resistivity of GST225 and GST147 during sequential current sweeps at various ambient temperature**

Fig. S2 illustrates how the resistivities of GST225 (Fig. S2(a)-(c)) and GST147 (Fig. S2(d)-(f)) depend on each current sweep at various temperatures: room temperature, 100 °C, and 200 °C. As the temperature increased, the start point of the resistivity decreased for both GST225 and GST147 because additional thermal energy causes a decrease in resistivity. However, resistivity at high temperatures eventually follow the same resistivity curve of that at room temperature over a certain current density which is the corresponding point of resistivity. The resistivity of GST147 shows a drastic decrease in current and temperature compared to that of GST225. The resistivity of GST147 was even saturated to the minimum value at 200 °C. These results indicate that the resistivity of GST147 is much more sensitive to external stimulus, such as electric current and temperature, compared to GST225.

**3. Gradual decrease in the resistivity of GST225 during thermal annealing**

Fig. S3 shows the XRD results for GST225 under different annealing conditions. The HCP transition temperature is generally near 350 °C:^1^ the XRD results at 350 °C are shown in Fig. S3. However, even at 250 °C, the hexagonal phase was transformed by long-term annealing. Because a high level of energy is required to generate the new phase, phase transitions are kinetically slow processes and are sensitive to the temperature. Therefore, the continuous decrease in the resistivity of GST225 during isothermal annealing, as shown in Fig. 2(c), is closely related to phase transformation.

**4. TCR change for deducing MIT**

The behavior of resistivity near 0 K is important to distinguish insulator from metal. Fig. S6 shows the resistivity of current-stressed GST225 for temperature ranging from 150 to 373 K. This results show that the behaviors of resistivity near room temperature have constant slope upto 150 K, which is similar with the reported results of Ge-Sb-Te compounds.^2,3^ Although the resistivity behavior near 0 K was not observed, the tendencies of resistivity change on current-stressed GST were similar with annealed GST. (GST225 with current density below 1 MA/cm^2^ are same with insulating GST, and GST225 with current density over 1 MA/cm^2^ are same with metallic GST) Therefore, the current-stressed GST225 over 1 MA/cm^2^ which has positive relationship between resistivity and temperature is estimated to have a finite value of resistivity at 0 K. The exact confirmation of the metallic behavior through near 0 K will be discussed in the further study.

**Reference for supporting information**

1 Friedrich, I., Weidenhof, V., Njoroge, W., Franz, P. & Wuttig, M. *J. Appl. Phys*. **87**, 4130 (2000).

2 T. Siegrist, P. Jost, H. Volker, M. Woda, P. Merkelbach, C. Schlockermann, and M. Wuttig, *Nature Materials* 10 (3), 202 (2011)

3 P. Jost, H. Volker, A. Poitz, C. Poltorak, P. Zalden, T. Schafer, F.R.L. Lange, R.M. Schmidt, B. Hollander, M.R. Wirtssohn, and M. Wuttig, Advanced Functional Materials, *article in press* (2015)


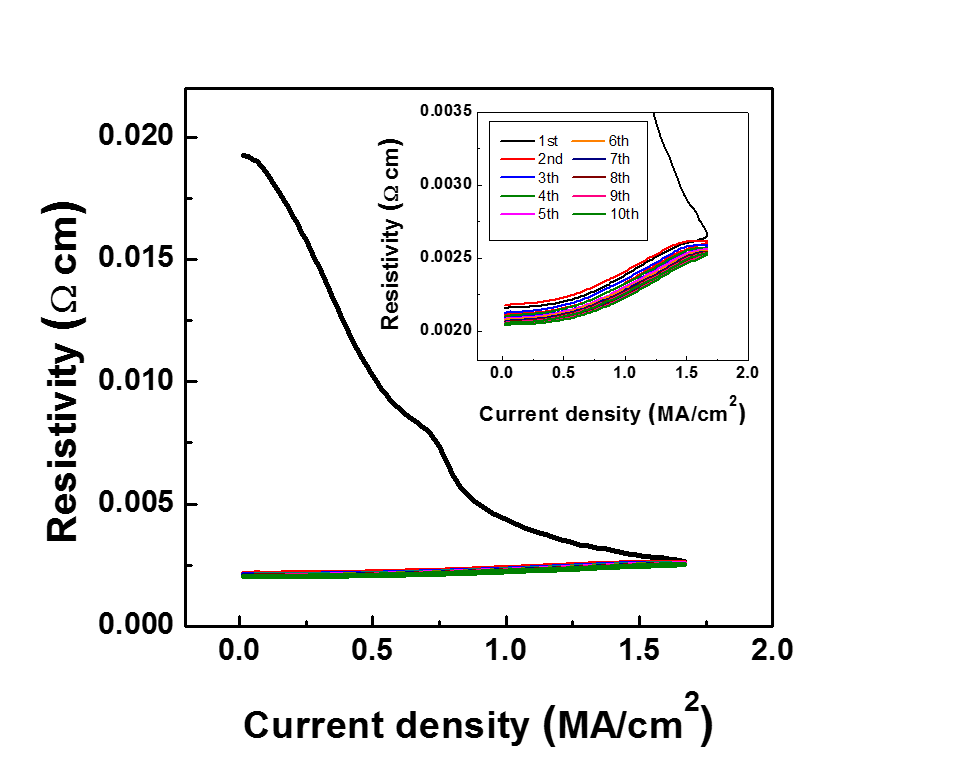


Fig. S1 Cyclic test for current sweep from 0 to 1.67 MA/cm^2^ with a fixed peak-current-density of 1.67 MA/cm^2^ at room temperature for GST225. The inset of Fig. S1 shows a resistivity during cyclic current sweep ranging from 0.0018 to 0.0035 Ωcm. An irreversible change in resistivity was completed during the first cycle of applied current.


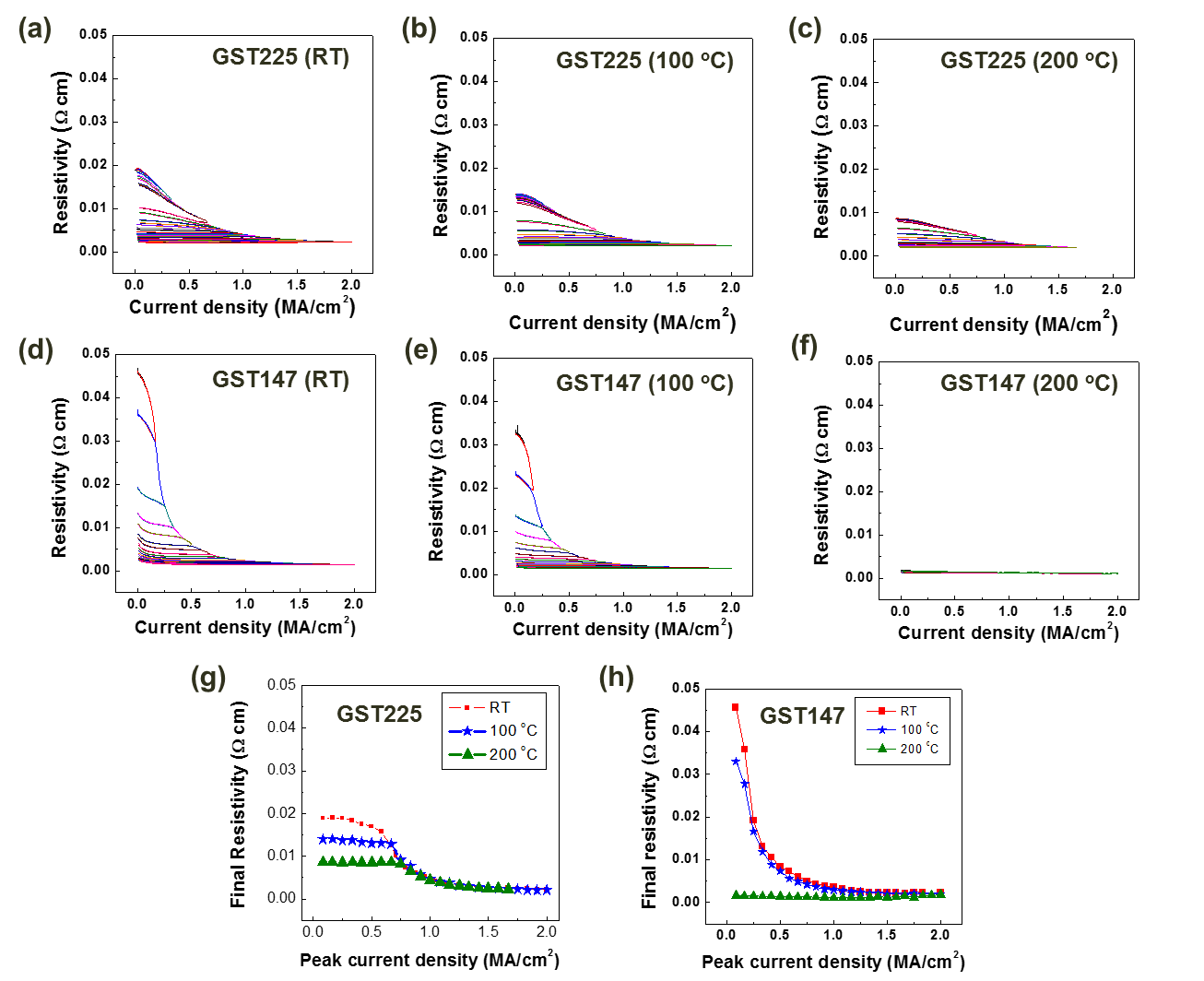


Fig. S2 (a)-(c) Resistivity change of GST225 for peak-current-densities ranging from 0.17 to 2 MA/cm^2^ in increments of 0.08 MA/cm^2^ at different temperatures: (a) room temperature, (b) 100 °C, and (c) 200 °C. (d)-(f) Resistivity change of GST147 for peak-current-densities ranging from 0.17 to 2 MA/cm^2^ in increments of 0.08 MA/cm^2^ at different temperatures: (d) room temperature, (e) 100 °C, and (f) 200 °C. Final resistivity of (g) GST225 and (h) GST147 according to the peak-current-density at various temperatures, based on the results from (a)-(c) for GST225 and (d)-(f) for GST147.


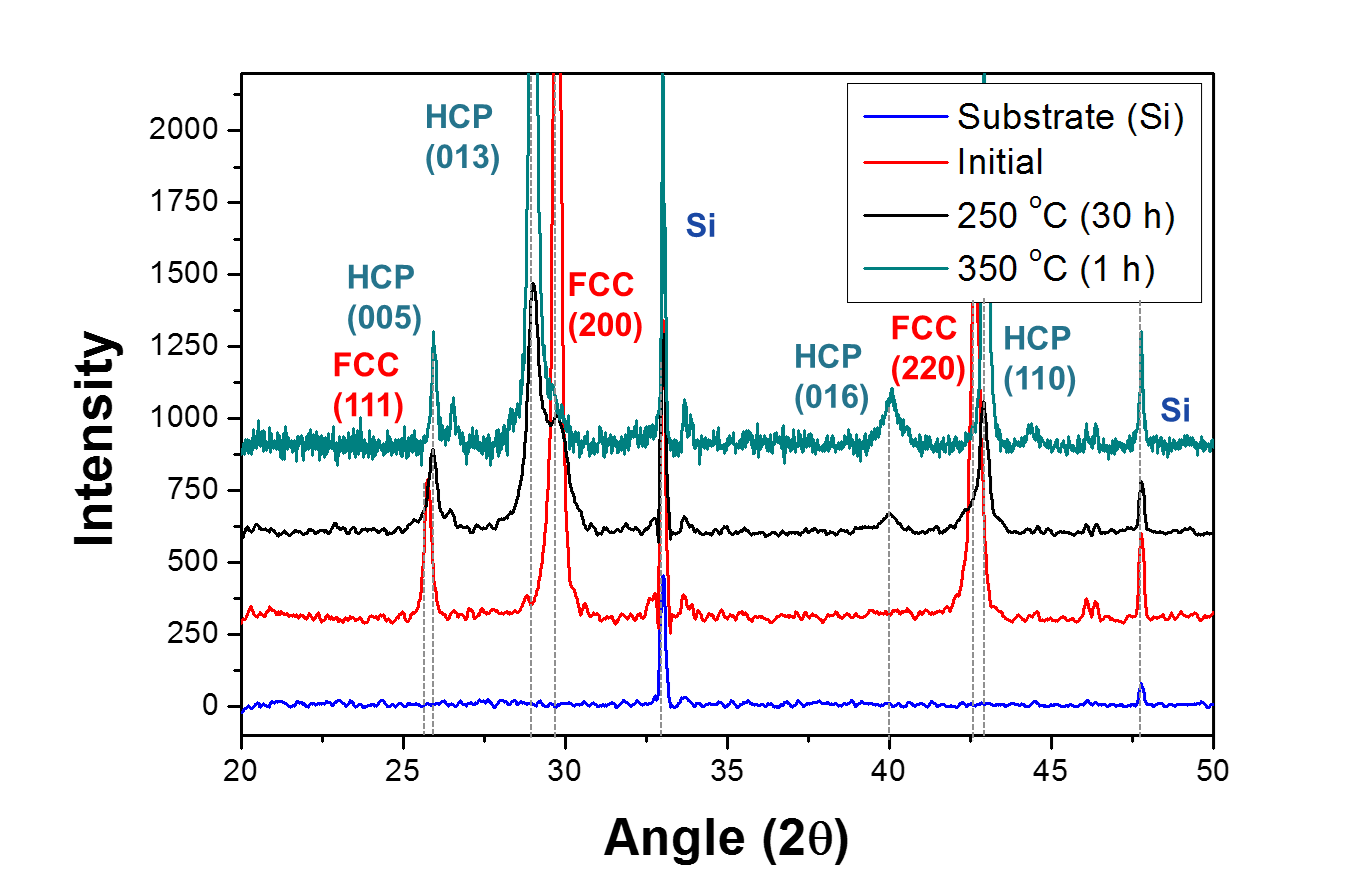


Fig. S3 XRD result of GST225 for different annealing temperatures: Si substrate (blue), pre-annealed GST225 (red), GST225 annealed at 250 °C for 30 hours (black), and GST225 annealed at 350 °C for 1 hour (green).


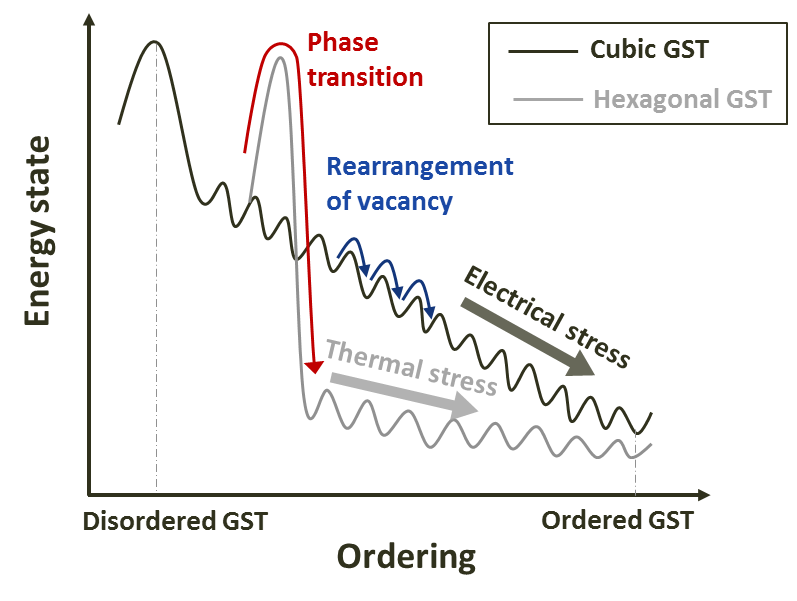


Fig. S4 The schematic diagram of the energy states according to the vacancy rearrangement in the cubic and hexagonal phases of GST. The energy state decreases with vacancy ordering, which is a stable state. The energy barrier for the phase transition from the cubic to the hexagonal phase is much higher than the energy barrier for the rearrangement of vacancies.


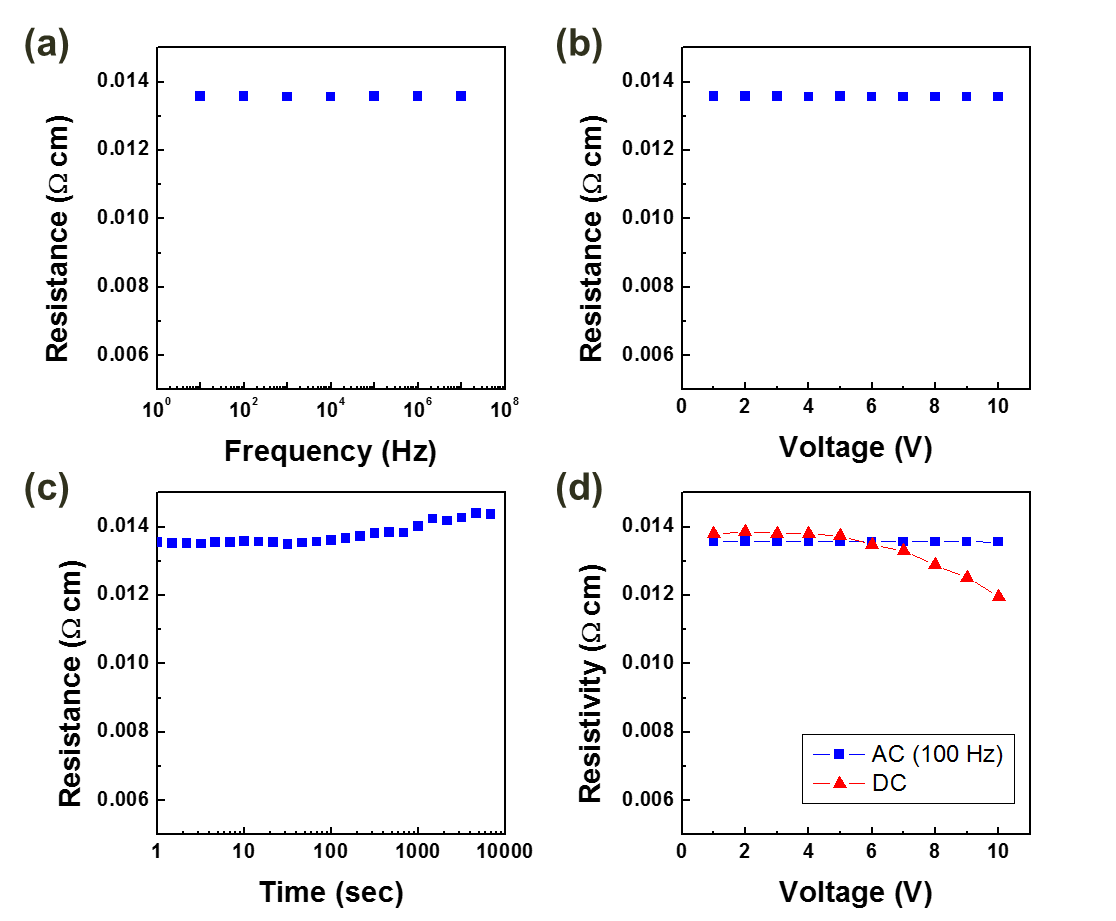


Fig. S5 Resistivity of GST225 affected by alternating current (AC) and direct current (DC). (a) Resistivity of GST225 according to AC frequency from 10^2^ to 10^7^ Hz with 1 sec of duration time and 10 V of voltage. (b) Resistivity of GST225 according to AC voltage from 1 to 10 V with 1 sec of duration time and 100 Hz of frequency. (c) Resistivity of GST225 according to AC time from 0 to 3 hours with voltage of 10 V and frequency of 100 Hz. (d) Resistivity of GST225 according to DC and 100 Hz of AC from 1 to 10 V with 1 sec of duration time.

Fig. S6 Resistivity of current-stressed GST225 for temperature ranging from 150 to 373 K. Conditions of current density were 0.67 MA/cm2 (black square), 1 MA/cm2 (red diamond), and 1.5 MA/cm^2^ (blue triangle).


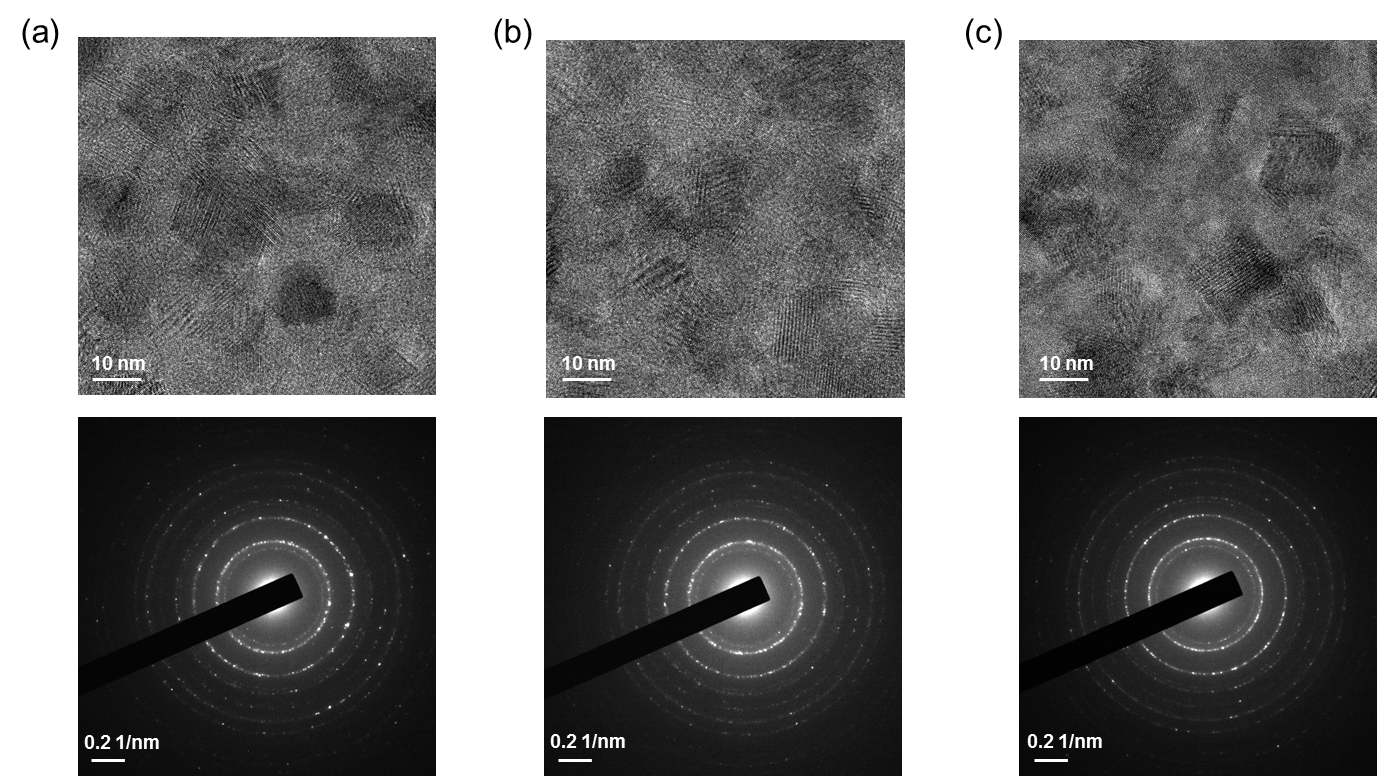


Fig. S7. HRTEM image and diffraction pattern at the position of (a) near cathode, (b) center, and (c) near anode in 1 MA/cm2-stressed GST225 line. Samples for TEM analysis were fabricated by Focussed ion beam (FIB). All results show polycrystalline cubic phase (FCC) independent with detecting position.
